# Determinants of myocardial conduction velocity: implications for arrhythmogenesis

**DOI:** 10.3389/fphys.2013.00154

**Published:** 2013-06-28

**Authors:** James H. King, Christopher L.-H. Huang, James A. Fraser

**Affiliations:** ^1^Physiological Laboratory, Department of Physiology, Development and Neuroscience, University of CambridgeCambridge, UK; ^2^Department of Biochemistry, University of CambridgeCambridge, UK

**Keywords:** conduction velocity, arrhythmia, sodium channel, gap junction, fibrosis

## Abstract

Slowed myocardial conduction velocity (θ) is associated with an increased risk of re-entrant excitation, predisposing to cardiac arrhythmia. θ is determined by the ion channel and physical properties of cardiac myocytes and by their interconnections. Thus, θ is closely related to the maximum rate of action potential (AP) depolarization [(*dV/dt*)_max_], as determined by the fast Na^+^ current (*I*_Na_); the axial resistance (*r*_a_) to local circuit current flow between cells; their membrane capacitances (*c*_m_); and to the geometrical relationship between successive myocytes within cardiac tissue. These determinants are altered by a wide range of pathophysiological conditions. Firstly, *I*_Na_ is reduced by the impaired Na^+^ channel function that arises clinically during heart failure, ischemia, tachycardia, and following treatment with class I antiarrhythmic drugs. Such reductions also arise as a consequence of mutations in *SCN5A* such as those occurring in Lenègre disease, Brugada syndrome (BrS), sick sinus syndrome, and atrial fibrillation (AF). Secondly, *r*_a_, may be increased due to gap junction decoupling following ischemia, ventricular hypertrophy, and heart failure, or as a result of mutations in *CJA5* found in idiopathic AF and atrial standstill. Finally, either *r*_a_ or *c*_m_ could potentially be altered by fibrotic change through the resultant decoupling of myocyte–myocyte connections and coupling of myocytes with fibroblasts. Such changes are observed in myocardial infarction and cardiomyopathy or following mutations in *MHC403* and *SCN5A* resulting in hypertrophic cardiomyopathy (HCM) or Lenègre disease, respectively. This review defines and quantifies the determinants of θ and summarizes experimental evidence that links changes in these determinants with reduced myocardial θ and arrhythmogenesis. It thereby identifies the diverse pathophysiological conditions in which abnormal θ may contribute to arrhythmia.

## Introduction

**Figure 1:**
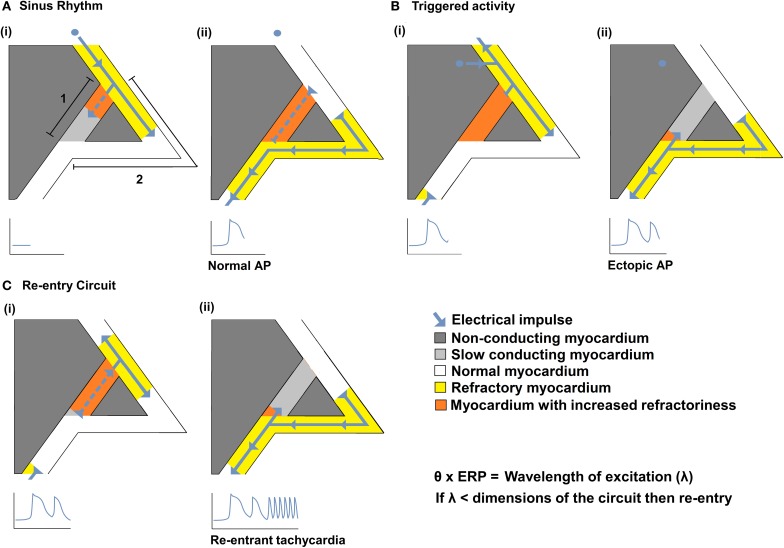
**The relationships between θ and re-entrant arrhythmia**. A diagram illustrating a typical re-entry circuit, consisting of a pathway of slow conducting myocardium [**A**(i) path 1, light gray] passing through non-conducting myocardium (dark gray), bordered by a second pathway of normal myocardium [**A**(i) path 2, white]. **(A)** An electrical impulse (blue arrow) originating from the SAN, (i) propagates along path 2 (white) and path 1 (light gray) pathways. As the impulse conducts, the myocardium becomes refractory (yellow in path 2 or orange in path 1) (ii) The impulse that travels along path 2 reaches the end of the circuit resulting in a normal AP. The impulse that conducts along 1 cannot exit the circuit as it collides with the refractory tissue of path 2. **(B)** An abnormal impulse originating from an ectopic focus is triggered immediately following the sinus impulse (i). It cannot conduct down path 1 which remains refractory; it therefore conducts along path 2. (ii) When the impulse reaches the distal end of path 2 it splits, conducting retrogradely along path 1 and orthogradely along path 2. **(C)** The impulse conducting retrogradely along path 1 then activates the beginning of path 2 (i) without the need of any further stimuli, thereby creating a self-perpetuating re-entrant rhythm (ii). Such re-entry is more likely to occur following reductions in conduction velocity (θ) and/or the effective refractory period (*ERP*) that reduce the wavelength of excitation (λ), given by the product of the θ and *ERP*, to values smaller than the dimensions of the available circuits.

Impaired myocardial action potential (AP) conduction can predispose to arrhythmogenesis through the formation of slow conducting re-entry circuits. Re-entry was first defined by Mines in the early twentieth century as a persisting electrical impulse that reactivates an area of previously activated myocardial tissue that is no longer refractory, resulting in a circus movement of activation (Mines, 1914). Subsequent studies suggest that sustained arrhythmia requires an ectopic AP triggering event to occur within such a substrate capable of generating self-sustaining re-entry processes (Mandapati et al., 2000; Zou et al., 2005). Thus, a triggered electrical impulse must enter a perpetuating electrical circuit containing a unidirectional conduction block along one of its two pathways. Such re-entry within the circuit is more likely to occur following reductions in conduction velocity (θ) and/or the effective refractory period (*ERP*). As shown in Figure 1, this could result in a reduction in the wavelength of excitation (λ), given by the product of θ and *ERP*, to values smaller than the dimensions of the available circuits (Mines, 1914; Wiener and Rosenblueth, 1946; Wakili et al., 2011). Alternatively if the site of slowed conduction is such that it prevents propagation of the triggered AP into the circuit, it may discourage re-entry.

This review describes the generation and propagation of the cardiac AP and defines the determinants of θ. It summarizes experimental evidence that links changes in these determinants with reduced myocardial θ and arrhythmogenesis, thereby identifying the diverse pathophysiological conditions in which abnormal θ may contribute to arrhythmogenesis.

## The generation of the action potential

Cardiac APs are time-dependent voltage waveforms that propagate along excitable tissues. AP generation arises from specific changes in membrane permeability resulting in a sequence of selective ion fluxes through their contained ion channels down their electrochemical gradients. Cardiac AP waveforms have been divided into five phases. The AP is initiated by transmembrane potential depolarization beyond an “activation threshold” at which inward currents exceed outward currents. This results in a Na^+^ influx through voltage-sensitive Na^+^ channels that show a regenerative increase to result in large depolarizing Na^+^ currents (*I*_Na_) (of order 400 μA μF^−1^). These generate the initial rapid (~400 V s^−1^) phase 0 upstroke of the AP. The maximum rate of AP depolarization [(*dV/dt*)_max_] in an isolated cell is thus directly proportional to the total ionic current across the cell membrane (*I*_*i*_) (Hodgkin and Katz, 1949).

As *I*_Na_ is the major transmembrane current in phase 0, (*dV/dt*)_max_ is therefore often used as an index of *I*_Na_. Having reached its peak amplitude, *I*_Na_ quickly inactivates in less than 1 ms, and requires an ERP to elapse before any further excitation. The succeeding brief rapid repolarization (phase 1) is additionally driven by the rapid activation and inactivation of K^+^ channels carrying the fast and slow transient outward currents, *I*_to,f_, and *I*_to,s_, respectively. This is then followed by a plateau phase (phase 2) during which inward Ca^2+^ currents (*I*_CaL_) through the L-type Ca^2+^ channels are balanced by *I*_NCX_, resulting from 3Na^+^/Ca^2+^ exchanger (NCX) activity, the ATP-activated K^+^ current (*I*_KATP_), and progressively activating *I*_ks_ and *I*_kr_,: the slow and rapid components of the delayed outward rectifying K^+^ current. Finally the outward currents, particularly *I*_Kr_, terminate the AP during phase 3 rapid repolarization. The resting membrane potential during the final, phase 4, electrical diastole, is primarily set by inward-rectifier currents (*I*_K1_), fixing the resting membrane potential close to the K^+^ equilibrium potential of about −80 to −90 mV.

The resting membrane potential persists until the next appropriately-initiated AP unless there is ectopic activity. Ectopic APs can be triggered by two types of afterdepolarization phenomena: early afterdepolarizations (EADs), or delayed afterdepolarizations (DADs). EADs are repolarization abnormalities characterized by oscillations in the membrane potential during phase 2 or 3 of the AP. They result from abnormalities in *I*_Na_ inactivation, reductions in the outward K^+^ currents (*I*_K1_, *I*_Ks_, and *I*_to_), or increases in inward *I*_CaL_, that then allow reactivation of *I*_Na_, a persistent late Na^+^ current, *I*_NaL_, or both, thereby compromising the net outward current required to repolarize the myocyte. In contrast, DADs are formed during phase 3 or 4 of the AP when Ca^2+^ released by an abnormal diastolic sarcoplasmic reticulum (SR) Ca^2+^ discharge is exchanged for extracellular Na^+^ via the NCX. Since NCX removes only 1 Ca^2+^ for every 3 Na^+^ entering, it causes a net inward current and depolarization of the cell. If the resulting afterdepolarization is large enough to displace the membrane voltage beyond the activation threshold, an extrasystole is induced.

## Action potential propagation and its determinants

The magnitude of *I*_Na_ also plays a major role in the subsequent propagation of the cardiac impulse to its neighboring cells. In a simple model of AP propagation, an axial current flows along a linear cellular structure, or cable, from one depolarized myocyte to its quiescent neighbor via intercellular channels known as gap junctions (Rohr, 2004). If this axial current is sufficient to depolarize the neighboring cell beyond its activation threshold, voltage sensitive Na^+^ channels will create transmembrane currents capable of propagating the AP. The axial resistance (*r*_a_) to such local circuit currents arises from the resistances of the cytosol and the gap junctions between adjacent cells. Thus, in addition to Na^+^ channels, gap junctions play a critical role in AP propagation and influence its velocity.

The determinants of θ can be identified using the nonlinear cable equation (Plonsey and Barr, 2007; Keener and Sneyd, 2009). This incorporates circuit elements each made up of a capacitance of unit fiber length, *c*_m_, (μF cm^−1^) in parallel with both a linear membrane resistance of unit fiber length *r*_m_ (kΩ cm) and nonlinear conductance elements responsible for individual ion channel properties. Together these generate a total current *i*_i_ in unit fiber length, *x*, as a function of time, *t*. Successive circuit elements are connected by elements reflecting cytoplasmic and gap junction resistances intervening between cells. These give rise to the effective intracellular *r*_a_ of unit fiber length, *r*_a_, In classical cable theory, *r*_a_ is assumed constant. The membrane potential, *V*, across any given capacitative element depends on the charging of its unit length by currents traversing the local membrane conductance elements, *i*_i_, as well as the axial current flow, *i*_a_, arising from neighboring regions along the length, *x*, of the element in question. Thus,

$$\frac{1}{r_{\text{a}}}\left(\frac{d^{2}V}{dx^{2}}\right)=c_{\text{m}}\left(\frac{dV}{dt}\right)+i_{\text{i}}$$

At constant conduction velocity θ = *dx/dt*, and so:

$$\frac{1}{\theta^{2}r_{\text{a}}}\left(\frac{d^{2}V}{dt^{2}}\right)=c_{\text{m}}\left(\frac{dV}{dt}\right)+i_{\text{i}}$$

This simplest version of the cable equation clearly identifies the key determinants of θ as *r*_a_, *c*_m_, and *i*_i_. However, several of its terms are interdependent, as will be discussed below, precluding its analytic solution. Furthermore, it is a stiff equation, requiring a good estimate of θ to be made before numerical solutions may be obtained (Jack et al., 1983) However, it is possible to derive simple relationships between θ and the parameters identified by this equation using a computer model of electrical conduction in a muscle fiber (Fraser et al., 2011). Although this model simulates skeletal rather than cardiac muscle, the insights that it provides into cable properties have general validity for any system in which fast sodium currents dominate the AP upstroke.

**Figure 2:**
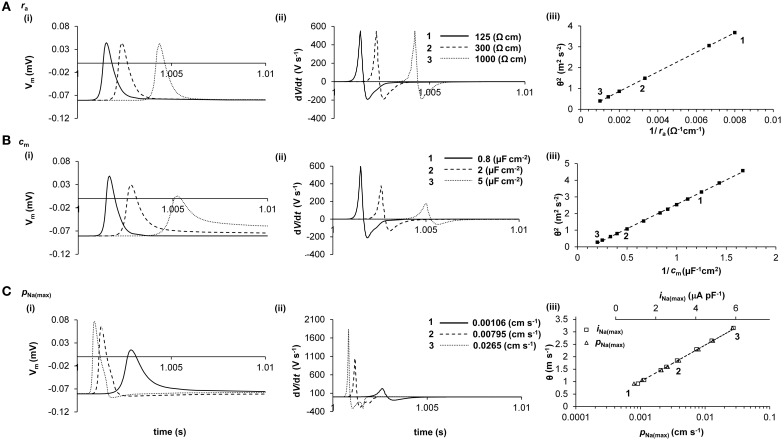
**Quantification of the determination of θ in a computer model**. The cable equation identifies *r*_a_, *c*_m_, and *i*_i_ as the determinants of θ, but does not clearly demonstrate the magnitudes of their influences. Computer modeling of a skeletal muscle fiber (Fraser et al., 2011) shows the influence of *r*_a_ (panel **A**), *c*_m_ (panel **B**), and *i*_Na(max)_ (panel **C**) on AP waveform (i), d*V/*d*t* (ii), and θ (iii). In panels i and ii, three representative APs are shown, each stimulated at 1s and recorded 2.5 mm further along the cable, such that increased times to the AP peaks denote slowed conduction. In panel iii, the velocities of these APs are labeled 1, 2, and 3. It will be noted that *r*_a_ influences θ but not AP waveform, whereas *c*_m_ and *i*_Na(max)_ each influence AP waveform and θ. Note that in panel **C**(iii), θ is plotted against both *i*_Na(max)_ (upper scale, squares) and *P*_Na(max)_ (lower scale, triangles). The simple quantitative relationships between these parameters that emerge from this analysis are given in the text.

Thus, Figure 2 demonstrates the empirical influences of *r*_a_, *c*_m_, and *i*_i_ upon θ and AP waveform in a computer model of AP propagation. It confirms that *i*_i_ is principally determined by *I*_Na_ during the AP upstroke, and demonstrates that *i*_Na(max)_ α log[*P*_Na(max)_] (*R*^2^ = 0.9965), where *P*_Na(max)_ is the maximum permeability of the fast Na^+^ channels. Several important relationships then emerge that allow measurements of the AP waveform to be used to investigate relative changes in *i*_Na(max)_ and *c*_m_.

First, (Figure 2A) *r*_a_ does not influence the AP waveform [Figure 2A(i)] and thus does not influence *dV/dt* [Figure 2A(ii)] or *d*^2^*V/dt*^2^ (not shown). Consequently, θ^2^ α 1/*r*_a_ (*R*^2^ = 1.0000) [Figure 2A(iii)] as predicted from the cable equation. This simple relationship emerges because *r*_a_ influences only the AP waveform as a function of distance, not as a function of time. The effect is similar if *r*_a_ is unevenly distributed, as in cardiac myocytes connected by gap junctions (data not shown). Simulations show that, although uneven distribution of *r*_a_ produces small increases in AP amplitude and (*dV/dt*)_max_ immediately before high resistance areas and small decreases in these parameters immediately after, θ and distance-averaged values of AP amplitude and (*dV/dt*)_max_ are influenced as for evenly-distributed increases in *r*_a_.

Second, (Figure 2B) increases in *c*_m_ influence the AP waveform [Figure 2B(i)], slowing the voltage excursions [Figure 2B(ii)] and producing a reduction in θ. Interestingly, θ^2^ α 1/*c*_m_ (*R*^2^ = 0.9996) [Figure 2B(iii)], as it appears in the cable equation, despite the influence of *c*_m_ on *dV/dt* and *d*^2^*V/dt*^2^ (not shown). These relationships have good empirical approximations: (*dV/dt*)_max_ α log(*c*_m_) (*R*^2^ = 0.9977) and (*d*^2^*V/dt*^2^)_max_ α 1/*c*_m_ (*R*^2^ = 1.0000).

Finally, (Figure 2C), the relationship between *i*_Na(max)_ and θ is difficult to derive from the cable equation because of the very large influence of *i*_Na(max)_ upon AP waveform [Figure 2C(i)] and (*dV/dt*)_max_ [Figure 2C(ii)] and *d*^2^*V/dt*^2^ (not shown). Nevertheless, the resultant empirical relationship for the range of values depicted in Figure 2C is straightforward: θ α *i*_Na(max)_ (*R*^2^ = 1.0000) [Figure 2C(iii)]. The AP waveform is influenced by *i*_Na(max)_ as follows: (*dV/dt*)_max_ $\alpha \sqrt{i_{\text{Na}(\text{max})}{}^{3}}$ (*R*^2^ = 0.9996); and (*d*^2^*V/dt*^2^)_max_ α *i*_*Na(max)*_^3^ (*R*^2^ = 0.9996).

The cable equation can be extended from geometrically well-defined cylinders to cardiac tissue consisting of a continuous network of electrically-coupled cells. In doing so the analysis above becomes extended to one that determines conduction velocity resulting from the match between current and load (Kucera et al., 1998). Such an approach has been used to describe the macroscopic passive electrical properties of cardiac muscle (Weidmann, 1970; Kléber and Riegger, 1987), the relationship between *dV/dt* and macroscopic (>1 mm) propagation (Buchanan et al., 1985) and changes in cell to cell coupling.

**Figure 3:**
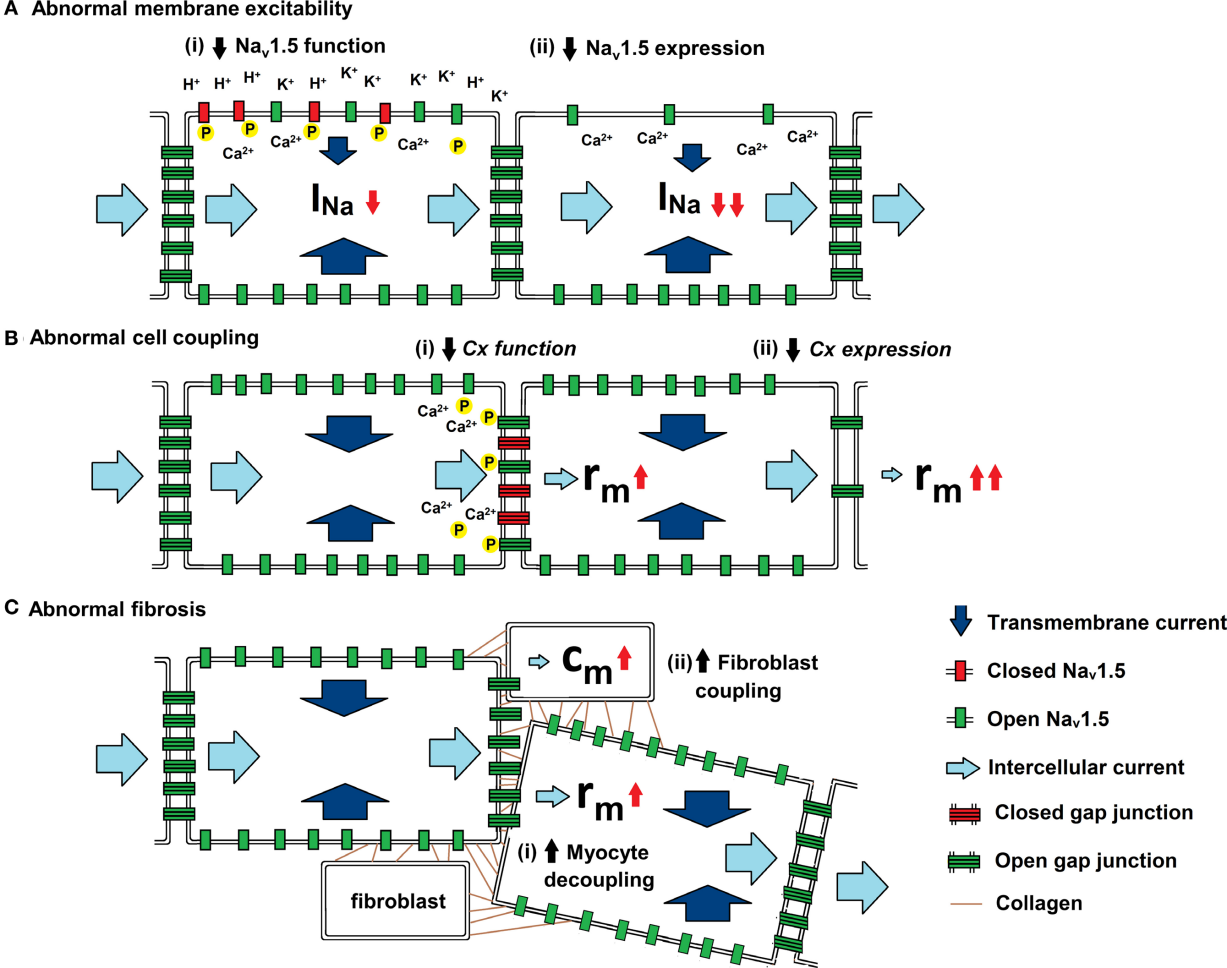
**Physiological influences on the determinants of θ**. Three diagrams illustrating the mechanisms by which (a) membrane excitability, (b) cell coupling and (c) fibrotic change influence current. Transmembrane current (dark blue arrow) enters through open Na_v_1.5 (green rectangle) and intercellular current (light blue arrow) passes through open Cxs (green ladder). **(A)** Abnormal membrane excitability results from reductions in either (i) Na_v_1.5 function through increases in extracellular [K^+^] and pH and by increases in [Ca^2+^]_i_ and phosphorylation, or (ii) Na_v_1.5 expression by mutations in *SCN5A* (Brugada syndrome) and through Ca^2+^ mediated down regulation of the channel. **(B)** Abnormal cell coupling results from reductions in either (i) Cx function through increases in [Ca^2+^]_i_ and dephosphorylation or (ii) Cx expression by mutations in either *CJA1 or CJA5* (idiopathic AF). **(C)** Abnormal fibrosis produces either (i) increased myocyte-myocyte decoupling, resulting in increased *r*_a_,. or (ii) Cx-mediated myocyte-fibroblast coupling, resulting in increased *c*_m_.

As summarized in Figure 3, experimental studies have suggested a range of mechanisms through which changes in AP propagation leading to increased arrhythmic tendency can take place. They have been attributed to alterations in Na^+^ channel and gap junction function, as well as to the consequences of fibrotic change. These could potentially alter the major determinants of θ: transmembrane current (*i*_i_), cell to cell coupling (*r*_a_), and cell capacitance (*c*_m_), outlined in the quantitative analysis above.

## The Na^+^ channel and its relationship to *I*_Na_

Na^+^ channels are transmembrane proteins responsible for a rapid, voltage-dependent, influx of Na^+^ ions. They are located within the surface and transverse (t)-tubular membranes (Cohen, 1996) mainly concentrating in the perinexus region near gap junctions (Lin et al., 2011; Rhett et al., 2012). Na^+^ channels consist of a principal α-subunit composed of four homologous domains each containing six, S1–S6, transmembrane segments. The function of the α-subunit is modulated by one or two associated ancillary β-subunits (Bezzina, 2001).

Several, Na_v_1.1, Na_v_1.3, Na_v_1.5, Na_v_1.6, α-subunits, are known to be expressed in the mammalian heart. Of these, Na_v_1.5, encoded by the *SCN5A* gene is the most abundant. It is a large 260 KDa glycosylated protein that forms the pore component of the channel and has very high selective permeability for Na^+^ (permeability ratio: Na^+^:K^+^ = 100.1) (Gellens et al., 1992; Wang et al., 1996). Na^+^ influx and the resulting current flow through the open Na^+^ channel (*I*_Na_) are responsible for the initial rapid (phase 0) AP depolarization and drives its propagation. It is consequently a key determinant of θ.

### Abnormalities in Na^+^ channel function

Abnormalities in conduction can arise from functional mutations in *SCN5A* that alter *I*_Na_ [Figure 3A(i)]. Of these the *SCN5a-1795insD* mutation is associated with an overlap syndrome with features of bradycardia, impaired conduction, LQT3, and Brugada syndrome (BrS) (Bezzina et al., 1999). Whilst mice homozygous for the mutation die *in utero*, the heterozygous *Scn5a*^1798*insD*/+^ mouse shows sinus node dysfunction, conduction slowing, and QT prolongation replicating the phenotype in humans (Remme et al., 2006).

Acquired abnormalities can also lead to changes in Na^+^ channel function. Abnormalities in AP depolarization were first described by Gelband and Bassett who recorded decreased values of (*dV/dt*)_max_ and depolarized resting membrane potentials in experimental models of heart failure (Gelband and Bassett, 1973). Subsequent studies have also associated heart failure with reductions in peak *I*_Na_ (Kuryshev et al., 1999; Ufret-Vincenty et al., 2001) through a number of mechanisms including reduced Na_v_1.5 glycosylation (Ufret-Vincenty et al., 2001). Pathophysiological reductions in Na^+^ channel availability have been additionally described during the acute phase of ischemia (Downar et al., 1977; Janse et al., 1986; Kleber et al., 1986; Kabell, 1989), tachycardia (Veenstra et al., 1987; Gaspo et al., 1997) and following treatment with class I anti-arrhythmic drugs (Sheldon et al., 1989).

Acute ischemia is pro-arrhythmogenic due to changes in intracellular and extracellular ionic concentrations, leading to reduced AP amplitudes, upstroke velocities (Downar et al., 1977; Janse et al., 1986) and conduction delays (Kleber et al., 1986; Kabell, 1989). Ischemic extracellular changes including: increases in [K^+^], decreases in pH, and hypoxia have been associated with modulation of Na^+^ channel function (Corr and Yamada, 1995). Microelectrode studies in hypoxic guinea pig papillary muscle demonstrated a decrease in (*dV/dt*)_max_ and depolarization of the resting membrane potential that was accentuated by increases in [K^+^] (Kodama et al., 1984). Subsequent studies in canine Purkinje fibers similarly showed a 8% decrease in θ in raised [K^+^] and a 4% decrease in θ following acidosis (Veenstra et al., 1987).

In addition to the extracellular effects, increases in intracellular cyclic adenosine monophosphate (cAMP) and cytosolic Ca^2+^ concentrations [(Ca^2+^)_i_] have also been reported in acute ischemia. Stimulation of β-adrenergic receptors is thought to produce adenylate cyclase-mediated increases in intracellular cAMP, leading to phosphokinase A (PKA) activation (Bers, 2002). Modulation of Na_v_1.5 by PKA occurs via phosphorylation at serine 525 and 528 within the DI–DII linker (Murphy et al., 1996). Following PKA activation, Na_v_1.5 redistributes to the plasma membrane of HEK293 cells (Hallaq et al., 2006). This may explain the increases in *I*_Na_ reported following PKA activation with dibutyryl cAMP in rabbit and canine myocytes (Matsuda et al., 1992; Baba et al., 2004).

However, further experimental studies variously show that treatment with isoproterenol either increases (Matsuda et al., 1992) or decreases (Ono et al., 1989; Schubert et al., 1990) *I*_Na_. Furthermore, when rabbit myocytes were treated with both a PKA inhibitor and isoproterenol, *I*_Na_ remained elevated suggesting that β-adrenergic stimulation produces an additional, PKA-independent modulation of the Na^+^ channel. Myocytes treated with a GTP analog and stimulatory G protein subunit showed increased *I*_Na_ implicating involvement of a G protein regulatory pathway (Matsuda et al., 1992).

In contrast to PKA activation, Ca^2+^-dependent activation of the protein phosphatase calcineurin has also been shown to strongly reduce *I*_Na_, This has been variously attributed to activation of protein kinase-C (PKC) or modulation of Na^+^ channel trafficking (Abriel, 2007). PKC also directly modulates Na_v_1.5 by phosphorylation at serine 1505 in the DIII–DIV inactivation gate, significantly reducing *I*_Na_ (Qu et al., 1996).

Changes in intracellular Ca^2+^ may also exert direct regulatory effects on the Na^+^ channel. Indeed the C-terminal region of Na^+^ channel constructs contain two Ca^2+^-sensitive regions: a calmodulin binding, IQ, domain, and a Ca^2+^ binding, EF-hand, motif (Wingo et al., 2004; Chagot et al., 2009). Thus, increases in CaMKII activity have been variously reported to increase (Aiba et al., 2010) or decrease (Wagner et al., 2006) peak *I*_Na_. Alternatively, intracellular Ca^2+^ has been shown to directly inhibit *I*_Na_ without affecting channel gating through a permeation block. Indeed, reductions in *I*_Na_ density and (*dV/dt*)_max_ have been reported following increases in [Ca^2+^]_i_ brought about by changing the Ca^2+^ concentration in the pipette solution in patch-clamped WT myocytes (Casini et al., 2009). Furthermore, reductions in (*dV/dt*)_max_, θ and increased incidences of arrhythmia have been shown in both homozygous gain of function *RyR2-P2328S* (*RyR2*^S/S^) and caffeine-treated WT hearts that have abnormal diastolic SR Ca^2+^ release (King et al., 2013b). Both immunohistochemical and biophysical studies subsequently attributed these abnormalities to the effects of Ca^2+^ homeostasis on *I*_Na_ function (King et al., 2013a).

Finally, treatment with class I anti-arrhythmic drugs modulates Na^+^ channel function. Thus, lidocaine, mexiletine, tocainide, and aprindine have been shown to block Na^+^ channels in the inactivated state whilst quinidine and disopyramide block the open channel (Kodama et al., 1986; Sheldon et al., 1989). Furthermore, lidocaine has been associated with impaired conduction and the induction of ventricular tachyarrythmias in experimental models (Anderson et al., 1990).

### Abnormalities in Na^+^ channel expression

Abnormalities in AP depolarization could also arise from an alteration in Na_v_1.5 expression [Figure 3A(ii)]. Knockout mutations in the *SCN5A* gene decrease *I*_Na_ and are associated with cardiac conduction diseases including Lenègre disease (Schott et al., 1999), BrS (Gussak et al., 1999), sick sinus syndrome (Benson et al., 2003) and atrial fibrillation (AF) (Laitinen-Forsblom et al., 2006; Ellinor et al., 2008).

Of these, BrS is associated with a high incidence of ventricular tachyarrhythmias and sudden cardiac death (Gussak et al., 1999). Although the exact pathophysiological mechanism is not yet known (Hoogendijk et al., 2010), mutations in 17 genes have been associated with BrS of which *SCN5A* mutations account for a significant proportion (Tan et al., 2001). Furthermore, BrS patients with Na^+^ channel mutations show significantly longer conduction intervals than those without *SCN5A* mutations (Smits et al., 2002). In addition, class I anti-arrhythmic drugs have been used to unmask the BrS ECG pattern by exacerbating pre-existing conduction abnormalities (Gasparini et al., 2003).

Conduction alterations in BrS have been studied using a murine model with knock-out mutations in *Scn5a* (Papadatos et al., 2002). The homozygous embryos die *in utero* with severe defects in ventricular morphogenesis. Heterozygous mice (*Scn5a*^+/−^) haploinsufficient for Na_v_1.5 show normal survival with several cardiac conduction defects including decreased atrial, ventricular and atrioventricular conduction and increased susceptibility to pacing-induced ventricular arrhythmias (Papadatos et al., 2002).

The expression of Na_v_1.5 has also been shown to be regulated by changes in intracellular ion concentrations, including Ca^2+^. Thus, Na_v_1.5 mRNA and Na_v_1.5 protein expression increased following treatment with the Ca^2+^ channel blocker, verapamil, and decreased following treatment with the Ca^2+^ ionophore calcimycin in rat cardiomyocytes (Offord and Catterall, 1989; Taouis et al., 1991; Duff et al., 1992). Similarly, Na^+^ current densities increased following elevations of [Ca^2+^]_i_ brought about by increased extracellular [Ca^2+^]. It decreased following reductions of [Ca^2+^]_i_ produced by BAPTA-AM in patch-clamped cultured neonatal rat myocytes (Chiamvimonvat et al., 1995).

Such changes in the expression of Na^+^ channels have been reported in experimental models of atrial tachycardia that are associated with increased [Ca^2+^]_i_ (Sun et al., 2001). Thus, atrial tachypacing decreased Na_v_1.5 mRNA, *I*_Na_, and θ over several weeks in canine models (Gaspo et al., 1997; Yue et al., 1999). In contrast, the development of AF does not further reduce atrial *I*_Na_ (Yagi et al., 2002) or Na_v_1.5 mRNA (van der Velden et al., 2000) in canine and goat models, respectively.

## Gap junctions and their relationship to *r*_a_

Gap junctions are non-selective membrane channels that form low resistance cell-to-cell connections that permit intercellular currents, as well as the transfer of ions, amino acids, and nucleotides. Their distribution within the cell membrane is tissue-specific and helps determine the magnitude and anisotropy of conduction. In general, cardiac cells express gap junctions near Na^+^ channels and at higher densities toward the ends of cells rather than their lateral margins, resulting in lower *r*_a_ and hence faster conduction in the longitudinal direction (Kumar and Gilula, 1996).

Gap junction channels are composed of a family of proteins known as connexins (Cx). Adjacent cells each contribute a hemichannel, made up of 6 Cxs, to the junction. There are 15 known Cxs defined by their molecular weight, each with different channel properties and gating mechanisms. Four main variants, Cxs 30.2, 40, 43, and 45, have been described in mammalian cardiac tissue (Davis et al., 1994). The type and distribution of these Cxs determines the properties of passive conduction throughout the heart. Cx43 is the most abundant and is expressed throughout the ventricular and atrial myocardium (Beyer et al., 1987), whilst Cx40 is limited to atrial tissue and the His Purkinje system (Gourdie et al., 1993a,b). Cx30.2 is only found in the atrioventricular node (AVN) (Kreuzberg et al., 2006) and Cx45 is variously reported to be expressed in the specialized conducting system (Coppen et al., 1998, 2001). Cx43 has a moderate conductance of ~110 pS and compared to other cardiac Cx is relatively insensitive to changes in transjunctional voltage (Moreno et al., 1994; Veenstra, 1996). Cx40 conductances are similar to Cx43, ~160 pS, but they have higher sensitivities to transjunctional voltage (Beblo et al., 1995; Bukauskas et al., 1995). In contrast, Cx45 has a much lower conductance at ~30 ps and is extremely sensitive to transjunctional voltage (Veenstra et al., 1994; Veenstra, 1996). These conductances are heavily regulated in healthy and pathophysiological myocardium. The resistance of gap junctions makes up approximately half of the longitudinal resistance in rat atria (Fry et al., 2012). Thus, the conductance of Cxs and therefore gap junctions help determine the magnitude of *r*_a_ and are an important determinant of θ.

### Abnormalities in gap junction function

Mutations in the genes encoding Cxs can change gap junction function [Figure 3B(i)] and thereby reduce cell to cell coupling. Loss-of-function somatic mutations of the *CJA5* gene that expresses Cx40 have been shown to result in idiopathic AF (Firouzi et al., 2004) and when combined with a *SCN5A* mutation, atrial standstill (Groenewegen, 2002). Heterozygous somatic missense mutations and polymorphisms within the gene's regulatory region have also been linked to conduction delays and AF (Gollob et al., 2006; Hauer et al., 2006). Other mutations of the Cx43 gene, such as *GJA1*, which affects phosphorylation sites, have been associated with cardiac structural abnormalities (Britz-Cunningham et al., 1995; Dasgupta et al., 2001) but without reported conduction abnormalities.

Acquired functional modifications of Cx [Figure 3B(i)] have been shown to arise during both heart failure and myocardial ischemia. Heart failure is associated with increased c-Src tyrosine-mediated tyrosine phosphorylation of Cx43 leading to decreased conductance (Toyofuku et al., 1999), conduction abnormalities and arrhythmia (Laurita et al., 2003).

During ischemia there are pathological decreases in the conductance of gap junctions following increases in [Ca^2+^]_i_ (Smith et al., 1995; De Groot et al., 2001), intracellular acidification (Yan and Kléber, 1992) and through changes in catecholamine-induced increases in cellular cAMP, which in turn modulate levels of phosphorylation. Acute increases in [Ca^2+^]_i_ occur in ischemic rabbit models (Dekker et al., 1996) and are associated with gap junctional uncoupling (De Mello, 1975; Smith et al., 1995) and decreased conductance (Kirchhoff et al., 1998; Gutstein et al., 2001). Such changes result in conduction slowing and conduction block (Dekker et al., 1996) that is exacerbated by increases in intracellular pH (Kleber et al., 1986). Myocardial ischemia also causes Cx43 to rapidly dephosphorylate (Huang et al., 1999; Beardslee et al., 2000) leading to its lateralization, transfer from the intercalated disks to intracellular pools and electrical uncoupling (Smith et al., 1991; Matsushita et al., 1999; Beardslee et al., 2000; Lampe et al., 2000). Dephosphorylation of Cxs may also be involved in lateralization of gap junctions resulting in conduction abnormalities in AF (Dobrev et al., 2012). However, other studies have reported that protein kinase C-dependent phosphorylation of Cx43 at serine 368 is associated with decreased gap junctional communication (Lampe et al., 2000) and conductance (Moreno et al., 1994; Kwak et al., 1995).

### Abnormalities in connexin expression

Abnormalities in AP propagation also arise from changes in the total number of Cxs [Figure 3B(ii)]. Such abnormalities have been extensively modeled experimentally by using genetically modified mice with altered Cx 40, 43, and 45 expression.

Cx45 knockout mice (Kumai et al., 2000) show normal contraction with atrioventricular conduction block and die *in utero* at day 10, consistent with studies showing Cx45 is uniquely expressed in the atrioventricular canal (Coppen et al., 1999, 2001). The homozygous Cx40 knockout mouse model shows slowed conduction and a partial atrioventricular block (Leaf et al., 2008); however arrhythmia was only observed in one study (Kirchhoff et al., 1998). The homozygote Cx43 knockout mouse (Cx43^−/−^) dies from neonatal pulmonary outflow obstruction (Reaume et al., 1995) but electrocardiography and optical mapping have been used successfully to measure θ in mice haploid insufficient for Cx43 (Cx43^+/−^). The first of these studies suggested slowed conduction (Guerrero et al., 1997) but later work contradicted these findings, showing no significant change in θ (Kirchhoff et al., 2000; Thomas et al., 2003). However, ischemic Cx43^+/−^ hearts have shown conduction abnormalities and higher incidences of spontaneous ventricular arrhythmias compared to WT (Lerner et al., 2000).

A cardiac-restricted homozygote knockout of Cx43 model was used to prevent the neonatal lethal structural defects in Cx43^−/−^. Such mice have a 90% reduction in Cx43 with normal heart structure and contractile function. Epicardial optical mapping showed that both longitudinal and transverse ventricular θ was reduced by 40–50%. In addition, Cx43 conditional knockout mice had high incidences of spontaneous ventricular arrhythmias and sudden cardiac death (Gutstein et al., 2001). These latter findings were supported by recent modeling suggesting that in non-ischemic conditions a 90% reduction in gap junctions is required to decrease θ by 50% (Jongsma and Wilders, 2000; Spach et al., 2000).

Interestingly, recent studies have proposed an additional, non-canonical method of cardiac conduction whereby the intercellular transfer of charge does not only occur by the passive flow of current through gap junctions. These studies suggest a role for extracellular space in modulating θ (Veeraraghavan et al., 2012) and that connexins actively augment current propagation by the accumulation of functional sodium channels at the perinexus of the intercalated disc and by the maintenance of narrow intercellular distances (Agullo-Pascual and Delmar, 2012; Rhett et al., 2013). Such a mechanism is supported by experimental evidence in cardiac restricted Cx43^+/−^ knockout mice that associates reduced Cx43 expression with reduced Na_v_1.5 expression and *I*_Na_ (Jansen et al., 2012). These findings highlight one of the limitations of using simple cable theory and help explain why decreases in connexin expression that are classically thought to increase *r*_a_ do not result in proportional decreases in θ.

Acquired changes in Cx expression have also been associated with a number of cardiac conditions including ventricular hypertrophy, heart failure and AF. During the early stages of ventricular hypertrophy there were increases in Cx expression and myocyte coupling. Thus, neonatal rat ventricular myocytes cultured in cAMP (Darrow et al., 1996) or angiotensin II (Dodge et al., 1998), both mediators of hypertrophy, showed increases in Cx43 expression and θ. More recent studies have shown a 100% increase in Cx43 expression and resulting θ in neonatal ventricular myocytes cultured with a mechanical load (Zhuang et al., 2000). θ typically increases in the acute phases of hypertrophy due to both increases in Cx43 expression and cell size that decrease intercellular and longitudinal resistance, respectively (Wiegerinck et al., 2006). Over prolonged periods of hypertrophy θ starts to decrease, as seen in chronic arrhythmic conditions (Cooklin et al., 1997) where Cx43 shows 25–50% downregulation (Kaprielian et al., 1998). The expression of Cx43 is down-regulated in experimental (Akar et al., 2004; Ai and Pogwizd, 2005) and clinical (Dupont et al., 2001; Kostin et al., 2004) studies of heart failure through recruitment of mitogen-activated protein kinase C-Jun NH2- terminal kinase (Petrich et al., 2002). Clinical heart failure studies showed Cx45 (Yamada et al., 2003) and Cx40 (Dupont et al., 2001) are up-regulated, possibly as a compensatory mechanism.

There are inconsistent clinical and experimental reports regarding changes in Cx expression during atrial tachyarrhythmias such as AF. Thus, experimental studies show up regulation of Cx43 in an atrial tachypaced canine model (Elvan et al., 1996; Sakabe et al., 2004), increased heterogeneity of Cx40 expression in tachypaced goat models with no changes in overall Cx expression (van der Velden et al., 1998). Whilst clinical studies support experimental findings of increased Cx expression on lateral cell surfaces (Smith et al., 1991; Polontchouk et al., 2001; Kostin et al., 2002) and increased heterogeneity (Dupont et al., 2001; Kostin et al., 2004) there is no consensus on changes in overall Cx expression. Studies show both increases (Dupont et al., 2001; Polontchouk et al., 2001) and decreases (Kostin et al., 2002; Nao et al., 2003) in Cx40 and Cx43 expression. However, conduction abnormalities are consistently described in experimental (Gaspo et al., 1997) and clinical studies of AF (Konings et al., 1994).

Such findings suggest changes in gap junction conductance do influence θ and arrhythmic susceptibility but only when the majority of gap junctions are impaired. Instead, reductions in membrane excitability and anatomical disruption are likely to have greater roles in most pathologies.

## Fibrosis and its relationship to *r*_a_ and *c*_m_

Fibroblasts make up 75% of the cells in the myocardium (Banerjee et al., 2006). Indeed, sequential contraction of the atria and ventricles is dependent on the annulus fibrosus cordis to electrically insulate the two structures. In normal wound healing they undergo apoptosis after having produced a mixture of cross-linked collagen and other extracellular matrix (ECM) (Gurtner et al., 2008).

However, in certain fibrotic conditions such as myocardial infarction and cardiomyopathy, fibroblasts do not apoptose and instead proliferate, migrate, and differentiate into myofibroblasts (Weber et al., 1994). Fibroblast and myofibroblasts then continue to increase ECM deposition between layers of neighboring cardiomyocytes unabated (Manabe et al., 2002). This results in altered tissue architecture that in turn reduces myocyte-myocyte coupling as well as results in the formation of fibroblast-myocyte couplings (Camelliti et al., 2004). Such changes could in turn alter *r*_a_ or *c*_m_, respectively slowing conduction.

### Disruption of cell-to cell coupling and alterations in *r*_a_

When healthy myocardium is disrupted by insulating connective tissue, the current provided by cells preceding the disruption may disperse along alternative branches, giving rise to a local conduction delay. Such current to load mismatches are found in fibrotic myocardium (Spach et al., 1988; De Bakker et al., 1993) and have been shown to impair conduction (Mendez et al., 1970; Spach et al., 1982).

Experimental canine models of fibrosis have shown how the deposition of insulating ECM and the decoupling of myocytes [Figure 3C(i)] lead to the interruption of both longitudinal and transverse cardiomyocyte-bundle continuity, producing zigzag conduction patterns (Spach and Boineau, 1997; Burstein et al., 2009). Such changes lead to conduction delays that predispose to re-entry arrhythmias in both experimental (De Bakker et al., 1993) and clinical studies (Spach, 2007).

### Coupling of cardiomyocytes to fibroblasts and alterations in *c*_m_

Fibroblasts have been shown to electrically couple with cardiomyocytes through Cx43 and Cx45 binding [Figure 3C(ii)] (Camelliti et al., 2004; Chilton et al., 2007). Fibroblasts lack ion channels associated with excitation. They also have depolarized resting membrane potentials (Kamkin et al., 1999) and would therefore modulate the membrane potential of the myocytes to which they are coupled, potentially inactivating Na^+^ channels. In addition, the coupling of fibroblasts to myocytes produces a net increase in cell capacitance, *c*_m_ (Miragoli et al., 2006).

Both these features resulted in a slowing of conduction and re-entrant events in cardiomyocytes co-cultured with fibroblasts (Maleckar et al., 2009; Xie et al., 2009). The magnitude of these effects on conduction appeared to be determined by the number of fibroblasts coupled to each cardiomyocyte, with significant changes observed when 10–30 fibroblasts were coupled per cardiomyocyte (Jacquemet and Henriquez, 2008; Maleckar et al., 2009; Sachse et al., 2009).

### Murine models of fibrosis

Experimental studies in mice have supported the association between impaired conduction and fibrosis. However, most murine models of fibrosis have concurrent pathological processes that may additionally contribute to abnormal conduction, making interpretation of their findings difficult. Thus, mutations in sarcomere protein genes cause hypertrophic cardiomyopathy (HCM), a congenital condition associated with myocyte enlargement and increased myocardial fibrosis, heart failure, and arrhythmia (Lu et al., 2013). Experimental murine models that carry mutations in the α- myosin heavy chain gene, *MHC403*, show fibrosis, conduction abnormalities, and increased incidence of inducible arrhythmia (Berul et al., 1998). Mutations in *SCN5A* are associated with both BrS and Lenègre disease (Schott et al., 1999). The latter condition produces progressive cardiac fibrosis and conduction abnormalities that lead to complete atrioventricular block and episodes of cardiac syncope. *Scn5a*^+/−^ hearts demonstrate similar age-related fibrosis and deterioration in conduction resembling the clinical phenotype of both Lenègre's disease (Lenegre and Moreau, 1963) and BrS (Coronel et al., 2005). Finally, age-related upregulation of transforming growth factor-β1 (TGF-β1) associated with increased measures of fibrosis have been reported in *Scn5a*^+/−^ hearts (Hao et al., 2011).

TGF-β1 mediated myocardial fibrosis also directly plays an important role in atrial arrhythmogenesis, including AF (Nattel et al., 2005). Experimental studies suggest that mice with increased expression of TGF-β1 have higher incidences of AF and conduction abnormalities as a result of raised levels of atrial fibrosis (Verheule et al., 2004). Furthermore, atrial fibroblasts appear more sensitive to the actions of TGF-β than their ventricular counterparts (Nakajima et al., 2000). TGF-β1 polymorphisms are also thought to be involved in inducing congenital heart block as a result of fibrosis, leading to a predisposition to AF (Clancy et al., 2003).

## Summary and conclusions

This review defines the determinants of θ as *I*_Na_; *r*_a_ and *c*_m_ and summarizes the mechanistic evidence that links changes in these determinants with reduced myocardial θ and arrhythmogenesis. Thus, firstly, (*dV/dt*)_max_ is reduced by impaired Na^+^ channel function that arises clinically during heart failure, ischemia, tachycardia, and following treatment with class I anti-arrhythmic drugs. Such reductions also arise as a consequence of mutations in *SCN5A* such as those in Lenègre disease, BrS, sick sinus syndrome and AF. Secondly, *r*_a_ may be increased due to decreased gap junction coupling following ischemia, ventricular hypertrophy, and heart failure, or as a result of mutations in *CJA5* found in idiopathic AF and atrial standstill. Finally, either *r*_a_ or *c*_m_ could potentially be altered by fibrotic change through its effects upon decoupling myocyte-myocyte connections and through myocyte-fibroblast coupling. Such changes are observed in myocardial infarction and cardiomyopathy or following mutations in *MHC403* and *SCN5A* resulting in HCM or Lenègre disease, respectively. The review thereby identifies the diverse pathophysiological conditions in which abnormal θ may contribute to arrhythmogenesis. Such findings provide insight into the mechanisms of arrhythmogenesis in common arrhythmias not usually attributed to impaired conduction such as those associated with abnormal Ca^2+^ homeostasis.

### Conflict of interest statement

The authors declare that the research was conducted in the absence of any commercial or financial relationships that could be construed as a potential conflict of interest.
